# Case report: Immunotherapy inducing unexpected overall survival in choroidal melanoma: about a case

**DOI:** 10.3389/fonc.2024.1319792

**Published:** 2024-04-19

**Authors:** Youssef Elhaitmy, Soukaina El Anssari, Pierre Fournel, Nawfel Mellas, Wafa Bouleftour, Michele Lamuraglia

**Affiliations:** ^1^ Department of Medical Oncology, North Hospital, University Hospital of Saint-Etienne, Saint-Etienne, France; ^2^ Department of Oncology, Hassan II University Hospital, Fez, Morocco; ^3^ Department of Pulmonology and Thoracic Oncology, North Hospital, University Hospital of Saint-Etienne, Saint-Etienne, France; ^4^ Oncology Unit, Hopital Prive de la Seine Saint Denis, Le Blanc-Mesnil, France; ^5^ Sorbonne Université, CNRS, INSERM, Laboratoire d’Imagerie Biomédicale, LIB, Paris, France

**Keywords:** choroidal melanoma, hepatic metastases, enucleation, immunotherapy, case report

## Abstract

Choroidal melanoma (CM) is the most common malignant ocular tumor in adults. The current treatment of metastatic CM is limited by the intrinsic resistance of CM to conventional systemic therapies. Immunotherapy alone or in association with cytotoxic treatment became a realist option treatment. Advancements in molecular biology have resulted in the identification of a number of promising prognostic and therapeutic targets. Herein, we report a rare case of 36-year-old patient with metastatic CM who presented a good long response to treatment with double immunotherapy reaching 3 years of overall survival, which has never been described in the literature.

## Introduction

The choroid is the layer between the sclera and the retina, which is a part of uveal tract of the eye. Choroidal melanoma (CM) is a subtype of uveal melanoma (UV) (1). UM is the most common cancer of the eye and is the most common primary intraocular neoplasm in adults (1, 2). It represents 3% to 5% of all melanomas (1). CM is the most common primary intraocular tumor in adults but remains a rare tumor estimated between 5.1 and 9 cases per million inhabitants per year (3, 4). Bilateral involvement is exceptional, reported in 0.18% to 0.2% of cases, but it should not be overlooked because early diagnosis and treatment improve survival and visual prognosis (5–8). The main clinical sign is the decrease in visual acuity. The dome or biconvex lens appearance on ultrasound is frequently observed. Conservative treatment is most often proposed on both eyes, and the risk of iatrogenic visual loss remains significant (3). The mortality of CM has been extensively studied after enucleation, it is approximately 30% at 5 years and 50% at 10 years (3, 4). Indeed, CM is characterized by a high risk of essentially hepatic metastatic evolution. Enucleation is the most widely used surgical technique, and the use of immunotherapy is essential at the metastatic stage (9, 10). Encouraging results have been reported with the combination of immune checkpoint inhibitors with objective responses varying between 18% and 51.9% (11, 12). The median overall survival (OS) of patients with metastatic CM treated with immunotherapy is between 5 months and 7.6 months with an anti–PD-1: Programmed cell death protein 1 alone and between 15 months and 19.1 months with a combination of anti–PD-1 and anti–cytotoxic T-lymphocyte-associated protein 4 (11, 12).We present a case of a patient with liver metastatic CM with good evolution under immunotherapy.

## Case report

**Figure 1:**
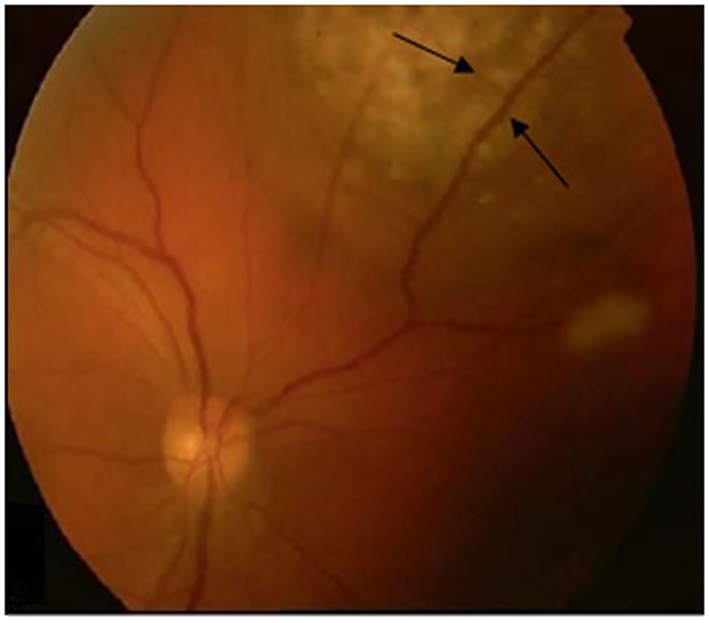
Fundus Oculi showing a pigmented uveal lesion in the right eye related to CM.

**Figure 2:**
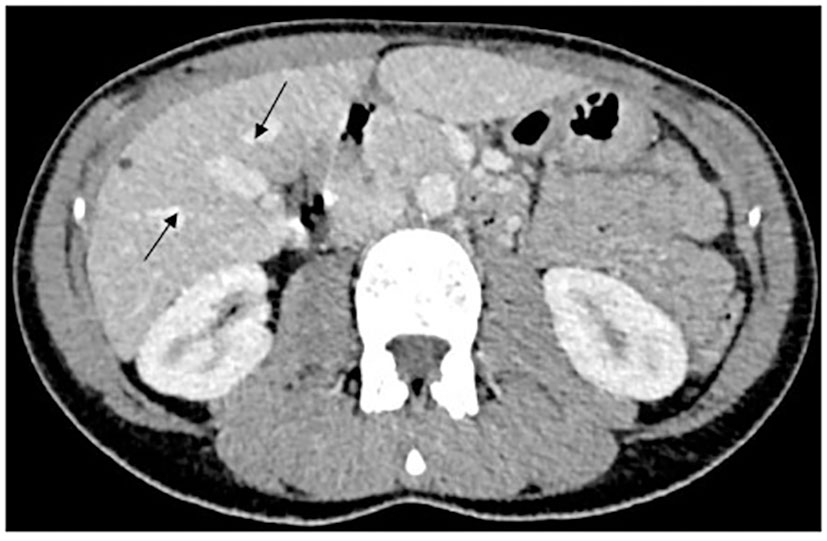
MRI hyper signal T2 showed two focal lesions of segments VIII and VI, and liver metastases from CM.

**Figure 3:**
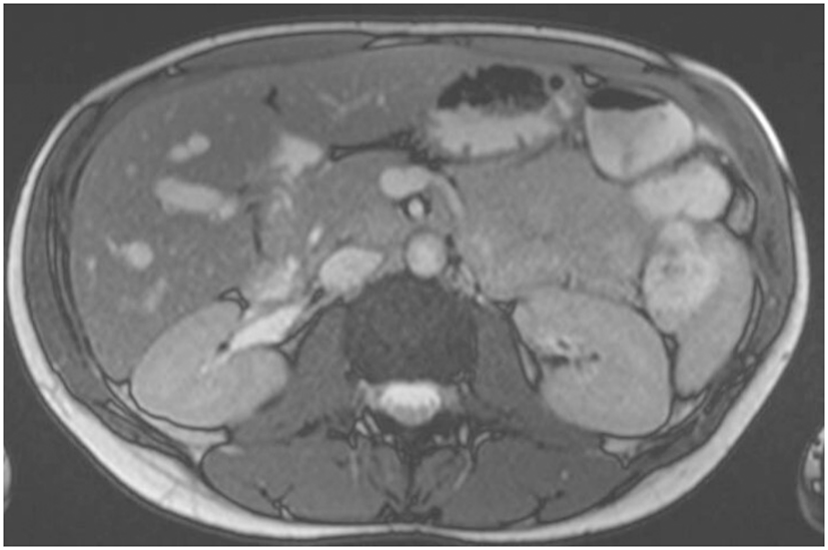
MRI showing complete response of liver metastases from choroidal melanoma.

A 36-year-old woman, without comorbidities and no smoking history, has been observed since 2019 by ophthalmologists for a melanotic lesion of the right eye (**Figure 1**). An ocular extension assessment was carried out, and no metastasis was evidenced through total body computed tomography (CT). Then, the patient benefited from a right enucleation, in which histological examination was in favor of a CM. Molecular biology revealed a genomic profile compatible with an intermediate-risk CM (D3/8g), without others genomics mutations. This lesion included an epithelioid cell, of 20 mm × 15 mm, an infiltration of the internal superficial layers of the sclera, and an invasion of the ciliary body, the iris, and the iridocorneal angle. No extra-scleral exteriorization, neither embolism, nor damage to the optic nerve were observed. Ten months later, during CT monitoring that showed liver’s suspicious images, the patient performed an MRI of the liver that confirmed the appearance of two focal lesions smoothed, 12 mm from segment VIII and 4 mm from segment VI. These lesions were in hyper T2 and hypo T1, had an enhancement in the arterial phase after injection, were rather homogeneous, and were suspicious. A biliary cyst of segment V was also observed (**Figure 2**). The patient received 4 months later a segmentectomy of segments VIII and VI. Pathological analysis confirmed metastasis of CM with in sano resection. Eight months later, another MRI was performed showing a progression with appearance of multiple lesions straddling between segments V and I and in segment VI. In view of the multiple hepatic metastasis, systemic treatment with immunotherapy combinations of NIVOLUMAB (1 mg/kg) and IPILIMUMAB (3 mg/kg) was initiated. After four cycles of NIVOLUMAB/IPILIMUMAB, the patient received six cycles of maintenance by NIVOLUMAB of 240 mg alone every 2 weeks for 3 months. The patient presented a complete response at the hepatic level after 3 months of maintenance treatment (**Figure 3**), without any adverse event. Given the good tolerance clinically and biologically a switch by NIVOLUMAB 480 mg / 4 weeks was continued. To date, the patient presents a complete response with total disappearance of liver metastases and an OS reaching 39 months along with a good tolerance of NIVOLUMAB.

## Discussion

The natural history of CM is punctuated by controversies intimately linked to the evolution of the guiding ideas of their treatments. In 1882, Fuchs advocated the importance of enucleation, an oncologically perfect procedure that removes tumors in their corneoscleral packaging protected from lymphatic dissemination (13). This operation, which had to be carried out as quickly as possible, gave rise to numerous diagnostic errors. In 1978, Zimmermann et al. put forward the hypothesis that enucleation was responsible for the appearance of CM metastases (14). These metastases would be due to the dissemination of malignant cells by manipulation of the eyeball during operations. The authors then recommended to perform enucleation only with great surgical precautions (14).

From 1962 to 1985, Gass studied the occurrence of metastases in CM. His findings showed that tumors that grew and are enucleated give metastases with the same frequencies as those that are enucleated from the outset. Moreover, there was little correlation between the cell type and the size of the tumor and its mode of growth, which is very variable. Therefore, two growth pattern hypotheses were proposed: exponential-type growth and Gompertzian-type growth, with variable doubling times during the life of the tumor (15).

In 1989, the Collaborative Ocular Melanoma Study started studying the comparative effectiveness of enucleation and brachytherapy of CM. The results of this study, published in 2001, showed that there is no significant difference in the occurrence of metastases according to the two modes of treatment. This suggests an early and treatment-independent metastatic spread (16, 17). It can be deduced that there is metastatic dissemination at a subclinical stage and that metastases can appear although the CM has been effectively treated. Rare cases of late metastases have been reported in the literature, sometimes up to 40 years after the discovery of the primary tumor (18, 19). Several hypotheses have been proposed to explain this supposed sleepiness of cancer cells: limitation of the growth of metastases by the absence of angiogenic activity and possible role of immune phenomena (20, 21). CM mainly spreads to the liver. This hepatic tropism remains poorly explained (4). Some authors have suggested the purely hematogenous nature of metastases and the role of chemokines receptors on the surface of tumor cells (21, 22).

Several factors have been described in the literature that can predispose to CM: blond hair, fair skin, inability to tan, and light eye color (4). A meta-analysis provided by Weis et al. investigated the association between host susceptibility factors and CM and found that light eye color, light skin color, and inability to tan were factors statistically significant (9, 10). This increased frequency may be associated with a lower presence of melanin in the choroid, which results in less protection against ultraviolet light and an increased risk of developing CM. The oculodermal melanocytosis is a congenital pigmentary anomaly characterized by slate gray pigmentation of the periocular skin, sclera, and uvea and constitutes an important risk factor for the development of CM. The risk for a Caucasian patient with ocular melanocytosis to develop CM is estimated to be 1 in 400. An association between CM and atypical cutaneous nevi has been established. Patients with atypical cutaneous nevi are 4.38 to 10.8 times more likely to develop ocular melanoma than the average population. Iris nevus is a risk factor for CM, although the rate of transformation of iris nevus into melanoma is not clearly understood. In a study of 170 patients with suspicious iris nevus, 5% of lesions showed clinical evidence of growth into CM at a mean follow-up of 5 years. In a recent study of 1,612 patients with iris nevus, only 3% of patients showed transformation of nevus to melanoma (10). By multivariable analysis, the features predictive of growth included age <40 years, hyphema, inferior tumor location, diffuse flat tumor configuration, and ectropion uveae. No evidence-based medicine indicates that occupational UV exposure is an independent risk factor for CM (9, 10). However, some studies suggest that it is an important risk factor for some patients occupationally exposed to artificial ultraviolet light. Various studies have explored the particular association between ultraviolet light exposure and occurrence of UM. However, published literature does not unequivocally implicate sunlight exposure as a risk factor for CM.

The types of mutations expressed in CM are essentially C (cytosine) > T (thymine) transitions. Moreover, somatic mutations in CM are rare and are attributed to deamination of methyl cytosines. This mutational rarity is not well elucidated. However, certain hypotheses are evoked such as the slow regeneration of stem cells or even very active repair mechanisms (23, 24). At the molecular level, two categories of mutations are distinguished: activation of membrane receptors coupled to Gap (GTPase-activating protein) proteins and mutation of BE (express base editor) genes (23).

CM is poorly chemosensitive (9, 10). Systemic chemotherapy uses deticene, carmustine, fotemustine, or cisplatin with objective response rates below 10% and a median survival of 6 months (25). Hepatic intra-arterial chemoembolization has also been developed. This consists in the administration directly in contact with the tumor of a cytotoxic agent (cisplatin) associated with embolization agents allowing to sequester the cytotoxic locally. The theoretical advantages of this technique are to reduce the systemic complications, to create local hypoxia allowing tumor necrosis and increase the local concentration of cytotoxic agent by a factor of 10 to 15, as well as its contact time with the tumor. The response rates vary according to the studies: up to 46% of responses with a median survival of 6 months to 11 months (26). CM has the particularity of expressing very little Programmed death-ligand 1 (PD-L1) on tumor cells and on tumor-infiltrating lymphocytes, but, in practice, the combination of dual immunotherapy remains essential in the absence of other therapeutic alternatives. Two phase II trials, CheckMate 401, evaluating the combination of NIVOLUMAB/IPILIMUMAB in patients with metastatic UM have reported ORRs of 11.5% and 18%, respectively (11, 12, 27). The median OS were 19.1 months and 12.7 months, respectively, and progression-free survival (PFS) were 5.5 months and 3 months, respectively, independently of PD-L1 expression (11, 12, 27). Comparative studies are needed to determine whether combination anti–CTLA-4/anti–PD-1 consistently improves outcomes in patients. In phase Ib/randomized phase II trial, percutaneous hepatic perfusion with melphalan with combination anti–CTLA-4/anti–PD-1 had improved promising results with a control of both hepatic and extrahepatic disease (28).

Among the limitations of our case report is the absence of PD-L1 status investigation. Therefore, all clinical trials in UM do not show correlation about efficacy and PD-L1 expression. A new option treatment came; Melphalan and Tebentafusp have been recently showing the efficacy in UM (29–31).

Melphalan is a member of the nitrogen mustard alkylating agent family that, in the SCANDIUM Trial, with isolated hepatic perfusion UV liver metastases, had showed increase in PFS of 4.1 months (29, 31).

Tebentafusp is a first-in-class bispecific fusion protein target the gp100 (a melanoma-associated antigen) through a high-affinity T-cell receptor, which redirects T cells to kill gp100-expressing tumor cells (33). The phase III (IMCgp100-202 trial) after 3 years of follow-up showed OS of 21.6 months in the Tebentafusp group vs. 16.9 in the control group, in HLA-A*02:01–positive metastatic UM patients (30).

In our case, we report a good complete response to NIVOLUMAB/IPILIMUMAB, followed for NIVOLUMAB maintenance, with an OS reaching 36 months vs. 19 months to 12 months described in the studies cited above. We also reached 33 months of maintenance with NIVOLUMAB alone nonstop, with excellent tolerance, without toxic effects. Regarding the duration of maintenance, only one study continued maintenance until progression and the other study stopped at 2 years (11, 12). As known, the major limitations of case reports are the lack of ability to generalize the validity of the study and, thus, the impossibility to establish cause–effect relationship. Therefore, further studies are needed to specify the duration of immunotherapy maintenance, evaluating the tolerance, as one of the ratios.

## Conclusion

CM is a rare tumor with a poor prognosis; despite optimal treatment of the primary tumor, metastasis occurs early with high probability. This case report shows an OS better than described in the literature reaching 39 months. Same researches are in progress to investigate the molecular characteristics involved in CM prognosis. More investigations are needed to identify a place of immunotherapy not only in metastatic but also in adjuvant or neoadjuvant indications, alone or in combination with cytotoxic treatment, to improve the prognosis of these tumors by offering early treatment for forms at high risk of relapse.

## Data availability statement

The original contributions presented in the study are included in the article/supplementary material. Further inquiries can be directed to the corresponding author.

## Ethics statement

Written informed consent was obtained from the individual(s) for the publication of any potentially identifiable images or data included in this article.

## Author contributions

YE: Conceptualization, Writing – original draft. SA: Investigation, Writing – review & editing. PF: Supervision, Validation, Writing – review & editing. NM: Validation, Writing – review & editing. WB: Supervision, Writing – review & editing. ML: Supervision, Validation, Writing – review & editing.
